# A Rare Presentation of Babesia‐Associated Splenic Infarction in an Immunocompetent Young Male With Mild Parasitemia

**DOI:** 10.1155/carm/5210024

**Published:** 2026-01-02

**Authors:** Jamal A. Anthony, Dejan Nikolic, Rosalie Pepe, Henry Fraimow

**Affiliations:** ^1^ Department of Infectious Disease, Cooper University Hospital, 1 Cooper Plaza, Camden, New Jersey, USA, cooperhealth.org; ^2^ Department of Medicine, Cooper Medical School of Rowan University, Camden, New Jersey, USA, rowan.edu; ^3^ Department of Pathology and Laboratory Services, Cooper University Hospital, 1 Cooper Plaza, Camden, New Jersey, USA, cooperhealth.org

## Abstract

Babesiosis is a vector‐borne protozoal disease primarily transmitted by the *Ixodes scapularis* tick, though it can also be transmitted through blood transfusions from infected donors. The illness can be asymptomatic or present with mild flu‐like symptoms. Still, in severe cases, it can lead to disseminated intravascular coagulation and severe hemolytic anemia, sometimes necessitating management in the intensive care unit. Traditionally, severe babesiosis has been linked to individuals over 50 years old, asplenia, and those who are immunocompromised. Notably, parasitemia levels greater than 10% are also associated with severe disease. However, we report a case of a young, immunocompetent male with severe babesiosis exhibiting severe hemolytic anemia and a rare complication of splenic infarction despite a low level of parasitemia. This case emphasizes that significant clinical complications, like spontaneous splenic infarction, can arise from low parasitemia levels, underscoring the need for heightened awareness of this potential outcome.

**Learning Points**

• Babesia‐associated splenic injury is a serious manifestation of babesiosis that does not always correlate with higher parasitemia levels; most cases are associated with low levels (< 5%).
• Coinfection with other vector‐borne diseases may amplify clinical severity.
• The patient’s clinical course should be the primary consideration in treating babesiosis, rather than the degree of parasitemia alone (except when the percent parasitemia is greater than 10%, in which case red cell exchange is recommended regardless of clinical presentation).
• For patients presenting with atraumatic splenic injuries or splenic injuries that are out of proportion to the trauma, it is essential to rule out babesiosis, as it is an emerging cause of atraumatic splenic injury. This helps to prevent treatment delays.
• Atraumatic splenic injury may be the only presenting sign, occurring without fever or systemic symptoms. Babesia‐associated splenic injury is a serious complication of the disease that does not require traditional risk factors for severe babesiosis, such as age over 50, immunocompromised state, or asplenia.

## 1. Introduction

Babesiosis is a vector‐borne illness caused by an intraerythrocytic protozoal parasite. It is most commonly transmitted by the *Ixodes scapularis* tick and, less commonly, by blood transfusion or organ transplantation [1–7]. There are many Babesia species. Most infect domestic and wild animals, with a few species causing human babesiosis [1–3]. Babesia microti, Babesia duncani, and Babesia divergens are known to cause human babesiosis [1–3]. It was first discovered in 1969 in an immunocompetent male from Nantucket Island, Massachusetts, who was found to have Babesia microti, giving it the name “Nantucket fever” [1, 2]. In the United States of America (USA), most cases are caused by Babesia microti [1–5].

Babesiosis is endemic in many states of the USA, particularly in the northeastern and midwestern regions, with a seasonal peak between the late spring and early fall (May–September) [1, 2]. The incubation period usually ranges from 1 to 4 weeks after the initial tick bite [1]. However, transfusion‐related cases tend to have a longer latency period, ranging from 1 to 9 weeks [1]. It can be asymptomatic in about 20% of patients [3]. Mild to moderate disease with symptoms often present with fever, chills, diaphoresis, headaches, myalgia, arthralgia, nausea, anorexia, and cough [1, 2]. Vannier et al. [1] also report that some patients may experience emotional lability, depression, hyperesthesia, sore throat, abdominal pain, vomiting, conjunctival injection, photophobia, and weight loss.

Spontaneous splenic injury (infarction or rupture) is a rare but severe complication of babesiosis that can result in an increased risk of morbidity and mortality if missed on initial presentation [2]. Here, we highlight that this complication of babesiosis can occur at any age, regardless of comorbidities or level of parasitemia.

## 2. Case Presentation

### 2.1. Case Description

**Figure 1:**
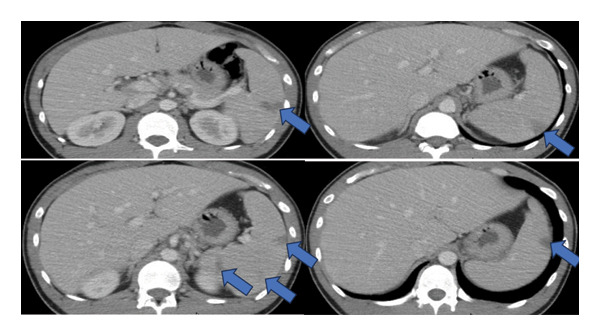
Still images from CT abdomen and pelvis from the patient’s first presentation to the referring hospital demonstrating splenic lesions, thought to be splenic infarcts.

A young, healthy male in his late 20s presented to a referring hospital in the early summertime with a worsening left upper quadrant pain, nausea, and vomiting, raising concerns about internal injury from a low‐level fall that had occurred 1 week before. He was hemodynamically stable and afebrile at that time. A computed tomography (CT) scan of the abdomen and pelvis suggested a splenic infarct or laceration (Figure 1). His symptoms improved after intravenous fluids and pain medication, and given that he was also hemodynamically stable, he was discharged with plans for outpatient follow‐up and repeat imaging.

**Figure 2:**
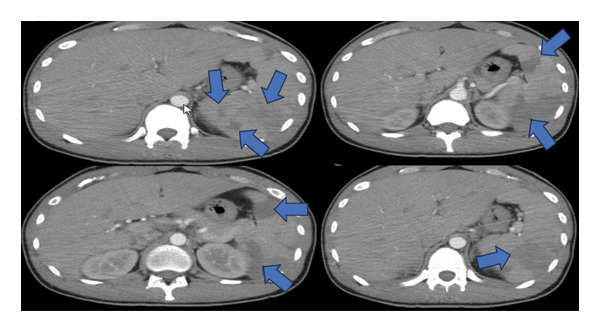
Still images from CT abdomen and pelvis from the patient’s 2^nd^ presentation to the referring hospital, 6 days after being discharged after his first presentation. These images demonstrate significantly enlarged splenic infarcts (blue arrows), splenomegaly, and hepatomegaly.

Six days later, he returned to the same hospital with increased fatigue, malaise, worsening left upper quadrant pain, decreased appetite, fever, and chills. A repeat complete blood count (CBC) showed that the hemoglobin level dropped from 12.4 g/dL to 8.4 g/dL (Table 1). Another CT indicated enlarged splenic infarcts with possible hematoma (Figure 2). He was admitted for further evaluation.

**Table 1:** Showing the trend of the patient’s CBC, hemolysis, coagulation, and anemia studies throughout his admissions.

Location/institution	Referring hospital visit 1	Referring hospital visit 2 (6 days later)		Tertiary institution 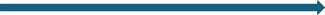								
Hospital day	1	1a	1b	1	2a	2b	2c	3	4	5	6	7
CBC results												
WBC (NR: 4.50–11.00 10^3^/μL)	5.18	8.37	8.12	7.52	7.00	7.30	7.71	7.55	7.59	7.86	6.74	7.01
Hemoglobin (NR: 14.0–18.0 g/dL)	12.4	8.4	7.9	7.2	7.0	6.9	7.1	6.6	6.5	7.9	8.0	8.9
Hematocrit (NR: 42.0%–52.0%)	36.2	27.1	24.7	22.8	22.4	22.4	22.2	20.7	20.7	24.9	25.9	29.4
MCV (NR: 80.0–94.0 fL)	84.6	84.7	87.3	87.4	87.5	88.2	87.7	88.5	90.4	89.9	93.2	95.8
MCH (NR: 27.0–31.0 pg)	29.0	27.8	27.9	27.6	27.3	27.2	28.1	28.2	28.4	28.5	28.8	29.0
MCHC (NR: 33.0–36.0 g/dL)	34.3	32.8	32.0	31.6	31.3	30.8	32.0	31.9	31.4	31.7	30.9	30.3
Platelet count (NR: 150–400 10^3^/μL)	156	169	181	179	204	213	196	216	213	230	275	338
Hemolysis, anemia, and coagulation studies												
Fibrinogen level (NR: 173–430 mg/dL)			633									
Ferritin (NR: 15–400 ng/mL)		> 1800					2366					
INR (0.8–1.2)		1.3	1.4	1.3								
Prothrombin time (NR: 9.9–13.5 s)		14.9	16.1	15.3								
Activated PTT (NR: 29.1–35.7 s)		30.1	23.2	29.7								
Haptoglobin (NR: 30–200 mg/dL)		< 15	< 10	< 10			< 10	< 10	< 10	< 10	< 10	
LDH (NR: 110–230 U/L)		701	749				720	906	789	744		
Reticulocyte count (NR: 0.5%–2.0%)		7.20	8.2									
Reticulocyte absolute count (NR: 0.0235–0.1220 10^6^/μl)		0.1886	0.2080									
Blood parasite smear % parasitemia			1.4%	1.3%			1.0%					

During hospitalization, he experienced fevers of up to 100.8 ^o^F, and his hemoglobin decreased from 8.9 to 7.9 g/dL, labs confirmed hemolysis (Table 1). His complete metabolic panel demonstrated normal renal function, mild transaminitis, and indirect hyperbilirubinemia (Table 2).

**Table 2:** Complete metabolic panel.

Location/institution	Referring hospital visit 1	Referring hospital visit 2 (6 days later)	Tertiary institution 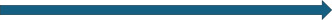					
Hospital day	1	1	1	2	3	4	5	6
Glucose (NR: 70–110 mg/dL)	87	112	96	111	140	117	114	141
BUN (NR: 9–23 mg/dL)	11	8	10	10	8	10	10	11
Creatinine (NR: 0.60–1.20 mg/dL)	0.93	0.87	0.92	0.77	0.73	0.76	0.76	0.84
Sodium (NR: 135–145 mmol/L)	138	133	139	135	132	136	134	138
Potassium (NR: 3.5–5.0 mmol/L)	3.9	4.3	4.5	4.6	4.4	4.5	4.0	4.2
Chloride, serum (NR: 96–108 mmol/L)	102	96	100	98	97	100	98	98
CO2 (NR: 22–28 mmol/L)	25	27	26	26	24	25	26	23
Albumin (NR: 3.8–5.3 g/dL)	4	3.9	3.3	3.3	2.9	3.1	3.6	3.8
eGFR								
> 59 mL/min/[1.73_m2]	> 60	> 60	116	125	127	126	126	122
Anion Gap (NR: 7–16 mmol/L)	11	10	13	11	11	11	10	17
AST (NR: 10–35 U/L)	36	58	52	48	64	109	65	50
ALT (NR: 6–45 U/L)	23	80	65	64	72	125	120	109
Alkaline phosphatase (NR: 39–117 u/L)	81	107	107	104	114	118	124	142
Bilirubin total (NR: 0.0–1.2 mg/dL)	2.6	1.6	1.9	2.1	1.3	1.2	0.9	0.8
Bilirubin direct (NR: 0.0–0.3 mg/dL)	0.5	0.5	0.8	0.9	0.4	0.4	0.4	0.2
Protein (NR: 6.0–8.5 g/dL)	7.8	8.4	7.5	7.4	6.9	7.2	7.5	8.3

*Note:* The patient had indirect hyperbilirubinemia and transaminitis. Hyperbilirubinemia resolved before discharge, and transaminase levels improved. Renal function remained preserved.

**Figure 3:**
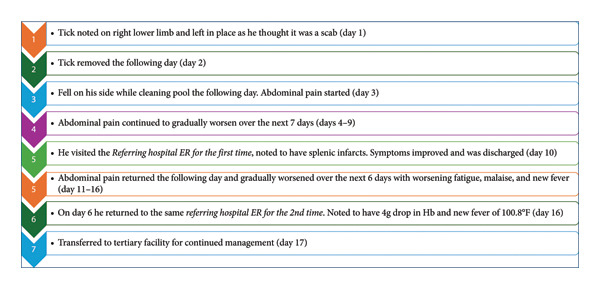
Timeline of events leading up to the transfer of the patient to our tertiary institution.

He was later transferred to our tertiary‐level facility for further management after spending one day admitted to the referring hospital (see Figure 3 for the timeline of events). He then revealed that he lived in a wooded area and had multiple recent tick bites, including one a week before his first visit (see Figure 3). He reported no rash or recent travel. Physical examination was notable for left upper quadrant tenderness with hepatosplenomegaly; there were no rashes, tick bites, or attached ticks.

### 2.2. Differential Diagnoses Considered and Management

The patient initially presented to a referring hospital with signs concerning for a traumatic splenic laceration following a fall. However, his stable vital signs and normal hemoglobin 1 week later made such an injury unlikely. Initially, the absence of fever and a normal white blood cell count made an infectious cause less likely, leading to his discharge.

Six days later, he returned with worsening abdominal pain, hemolytic anemia, and fever, expanding the differential diagnosis. Negative Coombs tests made autoimmune hemolytic anemia less probable. To rule out acute retroviral syndrome, 5^th^‐generation HIV testing was performed and was nonreactive. His physical examination and imaging showed no lymphadenopathy or signs of malignancy, such as longstanding unexplained weight loss, fevers, or night sweats. Given his fever, hemolytic anemia, elevated transaminases, and tick exposure, there was concern for tick‐borne illness.

Parasitic forms were observed during microscopic examination of a Giemsa‐stained thin peripheral blood film prepared for CBC with differential assessment. Considering his recent tick bite, residence in a forested area, and living in an endemic region, babesiosis became the leading diagnosis, especially in the absence of travel to a location where he could have been exposed to malaria.

Several distinguishing morphologic features of Babesia were identified: the intracellular ring stages forming tetrads (“maltese crosses”) in infected red blood cells (RBCs) (Figures 4(a), 4(b)) and extracellular forms of the parasite (Figures 5(a), 5(b)). Morphologically, on microscopy, possible infection with Plasmodium (Malaria) species was excluded. Based on these findings, the final diagnosis of Babesiosis was made with a 1.4% parasitemia level.

Figure 4(a) Parasite smear (Giemsa stain 1000×) showing Maltese Crosses (black arrows). (b) Intracellular *Babesia microti* (Giemsa stain 1000×).

**Figure:**
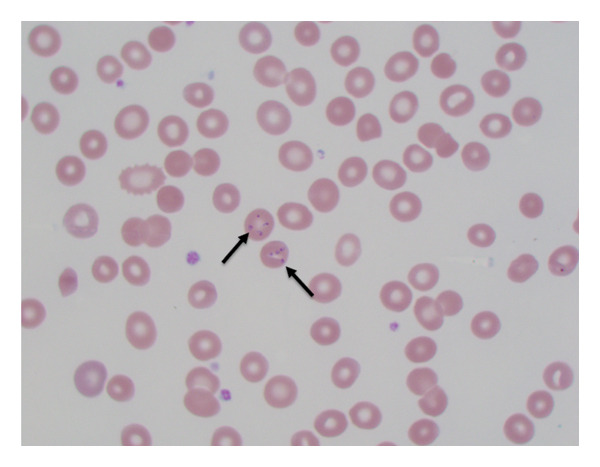
(a)

**Figure:**
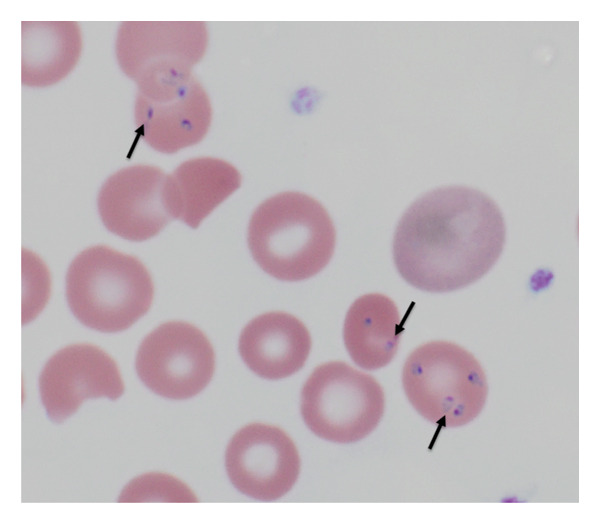
(b)

Figure 5(a) Giemsa‐stained thin blood film (1000×) showing extracellular forms of *Babesia microti* parasites (black arrows). Red arrows showing platelets. (b) Black arrow indicating extracellular *Babesia microti* (Giemsa stain 1000×). Red arrow showing platelet.

**Figure:**
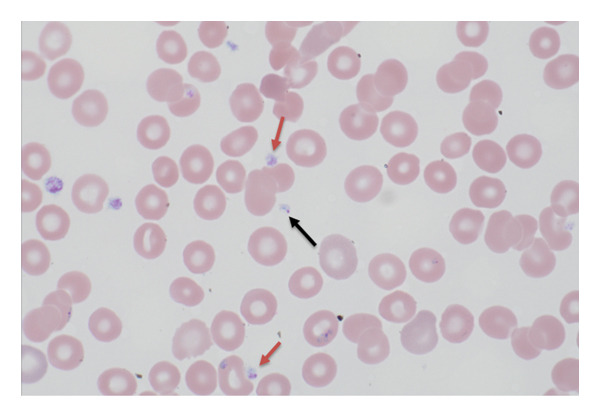
(a)

**Figure:**
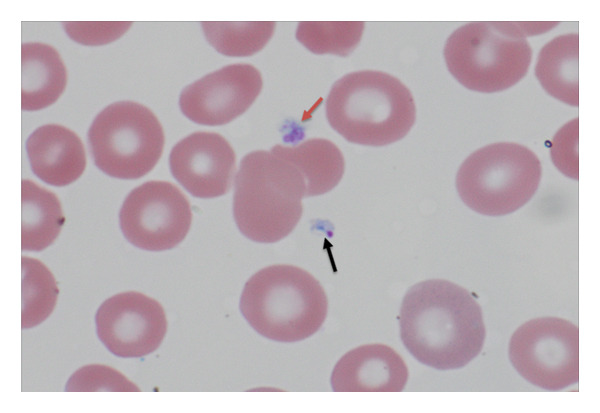
(b)

Considering coinfections with other vector‐borne infections transmitted by similar or identical vectors in endemic areas are not uncommon [8], they were also considered. The screening for coinfections revealed acute Lyme disease (IgM western blot positive; IgG immunoblot negative), while the testing was negative for Anaplasma phagocytophilum (no morulae visualized in peripheral blood neutrophils, and PCR using species‐specific primers was negative).

### 2.3. Treatment

At the beginning of his admission to our tertiary center, the patient had worsening hemolytic anemia even with the initiation of treatment on day one of admission (Table 1). He required a transfusion (days 3 and 4) of 2 units of packed RBCs to maintain his hemoglobin level above 7. As he continued treatment for his babesiosis, his hemolytic anemia stabilized by day 5, and his hemoglobin remained above 7 without the need for further transfusions. Our patient was classified as having mild parasitemia (percentage parasites being < 4%) but with severe clinical disease (severe hemolytic anemia < 10 g/dL, required hospitalization, and his splenic injury). He was treated with atovaquone (750 mg PO BID) + azithromycin (250 mg PO daily) regimen for 10 days. Lyme disease testing was positive, indicating coinfection. Doxycycline 100 mg PO BID was also prescribed (10 days) for coinfection with Borrelia, which was his only identifiable risk factor for severe disease.

### 2.4. Outcome and Follow‐Up

He remained stable. His abdominal pain and fever resolved by day 5, his splenomegaly improved, and his appetite returned. His hemoglobin also remained stable. As such, he was discharged to finish his course of antibiotics. The patient did well, with complete resolution of his symptoms and no further transfusions.

## 3. Discussion

### 3.1. Clinical Relevance

Our patient presented with fever, chills, severe abdominal pain, nausea, anorexia, fatigue, and malaise due to babesiosis, resulting in hospitalization. While most cases do not require hospitalization, babesiosis can lead to up to 21% mortality in hospitalized patients [1–3]. Historically, severe cases are typically observed in patients who are immunocompromised, aged > 50 years, and who have asplenia [1–18]. Additionally, Babesia divergens infection, compared to Babesia microti, babesiosis of any type with severe parasitemia (> 10%), as well as transfusion‐related babesiosis, have been linked to more severe disease [1, 2]. However, a growing body of evidence suggests that severe clinical manifestations of babesiosis, such as spontaneous splenic injury, can occur in immunocompetent young individuals with low levels of parasitemia. Therefore, it is crucial for clinicians to understand how to identify and provide appropriate treatment.

### 3.2. What This Case Contributes to the Literature

Table 3 shows a list of 20 cases of babesia‐associated splenic injury obtained from our literature review. Additionally, we found a systematic review by Dumic et al. [2] of 34 cases of babesia‐related splenic injury (which already included 17 of the 20 cases reported in Table 3). Our search identified three additional cases (cases 18–20), bringing the total to 37. Therefore, our case is the 38th documented instance. Of the 37 cases, most had low‐grade parasitemia < 5%, were all immunocompetent, and only 3 of 37 (8%) cases found were < 40 years old. Only one case reported by Abbas et al. [14] involved a patient < 30 years old. However, the 23‐year‐old patient described by Abbas et al. had severe parasitemia (30%), which likely contributed to his severe clinical disease and splenic injury.

**Table 3:** A list of reported babesiosis cases that have been associated with splenic complications and how each one compares to the patient described in this case report.

Case no.	Author	Year	Age	Comorbidities	Presenting symptoms	Immune status	Splenic pathology	Babesia species	Parasitemia level	Coinfection	Missed on initial presentation	Outcome
1.	Javed et al. [19]	2001	85 (M)	Hypertension	Fever, chills, malaise	Immunocompetent	Infarction	Microti	8%	Ehrlichiosis	**Yes**	Patient expired despite medical therapy
2.	Froberg et al. [15]	2008	56 (M)	Hypertension, cigarette smoking, and bilateral inguinal hernia repair status post laparoscopic surgery	Two week history of fever up to 103°F, chills, myalgia, and 10‐pound weight loss	Immunocompetent	Rupture	Microti	Not reported	Lyme disease	No	Splenectomy + medical management
3.	Kuwayama and Briones. [16]	2008	61 (M)	None	Fever, chills, headache, and malaise	Immunocompetent	Rupture	Microti	5%	Not reported	No	Splenectomy + medical management
4.	Florescu et al. [5]	2008	58 (M)	malaria in childhood	Fever, chills, myalgias, and left upper quadrant pain	Immunocompetent	Infarction	Microti	0.5%	Anaplasmosis	No	Medical management
5.	Florescu et al. [5]	2008	75 (F)	Colon cancer status postcompletion of chemotherapy 5 years prior and hypertension	Fever, night sweats, and fatigue	Immunocompetent but a history of being immunocompromised	Infarction	unknown	< 1%	No	**Yes, the patient was initially being worked up for malignancy as initial smears were negative. Treatment started 25 days after presentation. Course was complicated by aspiration pneumonia, sepsis multiorgan failure, and death.**	Patient demised despite medical management
6.	El Khoury et al. [10]	2011	36 (M)	None	Left upper quadrant pain and 2 weeks of fever and chills	Immunocompetent	Infarction	Microti	3%–4%	No	No	Medical management
7.	El Khoury et al. [10]	2011	70 (M)	Lyme disease 10 years prior	A 4 day history of malaise, fatigue, fevers, chills, and diffuse abdominal pain	Immunocompetent	Rupture	Microti	2%	No	No	Splenectomy + medical management
8.	Reis et al. [11]	2011	70 (M)	Not reported	Three day history of fever, nausea, and vomiting, and sudden onset of abdominal pain	Not reported	Rupture	Microti	Not reported	No		Selective splenic artery embolization
9.	Abbas et al. [14]	2011	23 (M)	None	Fever, chills, weight loss, malaise	Immunocompetent	Rupture	Microti	30%	No	**Yes**	Splenectomy + medical management
10.	Tobler et al. [17]	2011	54 (M)	Lyme disease	Left upper quadrant abdominal pain, fever of 102.3 °F, nausea, chills, night sweats, and dark urine	Immunocompetent	Rupture	Microti	3%	No	No	Splenectomy + medical Management
11.	Usatii et al. [12]	2014	54 (M)	None	Left upper quadrant abdominal pain for 1 week along with abdominal distention and subjective fevers and headache	Immunocompetent	Rupture	Microti	Not reported	No	**Yes**	Splenectomy + medical management
12.	Farber et al. [9]	2015	59 (F)	CAD, hyperlipidemia, and depression	A syncopal episode after 2 weeks of headaches, fatigue, chills, abdominal pain, nausea, and diarrhea	Immunocompetent	Rupture	Microti	0.9%	No	No	Splenectomy + medical management
13.	Al Zoubi et al. [18]	2016	72 (M)	Hypertension	Fever, chills, weight loss, and abdominal pain	Immunocompetent	Infarction	Microti	0.5%	No	**Yes**	Medical management
14.	Dumic et al. [6]	2018	79 (F)	Hypertension, coronary artery disease, and atrial fibrillation on warfarin	Left‐sided chest pain, extreme fatigue, and dizziness without any fever but she represented on postop day 10 with fevers and abdominal pain.	Immunocompetent	Rupture	Microti	1.6%	Lyme	**Yes: she presented without fever with splenic pathology, discharged, and represented with fever and then found to have babesiosis as the cause of her splenic injury.**	Splenectomy + medical management
15.	Blackwood et al. [7]	2018	51 (M)	Hypertension and atrial fibrillations	Syncope after a 5 day history of fever, chills, rigors, sweats, and general malaise with worsening abdominal pain	Immunocompetent	Rupture	Microti	0.9%	No	No	Splenectomy + medical management
16.	Blackwood and Binder [7]	2018	61 (M)	Hypertension and hyperlipidemia	Abdominal pain and 3 days of fever, chills, myalgias, weakness, and decreased appetite.	Immunocompetent	Infarction	Microti	0.44%	No	No	Medical management
17.	Gupta et al. [4]	2019	53 (M)	Tobacco use and cholecystitis status postcholecystectomy	Fatigue, fever, and abdominal pain	Immunocompetent	Infarction	Microti	1.5%	No	No	Medical management
18.	Sung et al. [3]	2021	46 (M)	None	Abdominal pain	Immunocompetent	Rupture	Microti	0.1%	Lyme disease	No	Splenectomy + medical management
19.	Sung et al. [3]	2021	51 (M)	Obesity status postgastric bypass surgery	Fever and left‐sided abdominal pain	Immunocompetent	Infarction	Microti	0.6%	Lyme disease	**Yes: Initial CT scan was unrevealing, and patient was discharged. He then returned with new hepatosplenomegaly, hemolytic anemia, acute splenic infarction.**	Medical management
20.	Mateja et al. [13]	2024	70 (M)	Hypertension, hyperlipidemia, BPH, asthma, gastroesophageal reflux disease, depression, anxiety, and schizoaffective disorder	Bilateral flank pain, sweating, nausea, and chills for 3 days. He reported having three syncopal episodes on the day of the presentation.	Immunocompetent	Rupture	Microti	13%	Not reported	**Yes, he had splenectomy, then was discharged. Returned after developing fever on POD 17. Pathology notified primary team of babesia seen on examination of spleen. He required plasmapheresis**	Splenectomy + medical management

*Note:* M (male) and F (female). The bold wordings emphasize cases in which the diagnosis was missed at initial presentation.

Abbreviations: BPH, benign prostatic hyperplasia; CAD, coronary artery disease; POD, postoperative day.

In contrast, our patient was an immunocompetent male in his late 20s with low‐level parasitemia (1.4%) with associated splenic infarction and severe hemolytic anemia, both known serious complications of babesiosis. Hence, we present a notable case of the youngest immunocompetent patient with babesia‐associated splenic injury in the setting of low parasitemia levels (< 4%). Our findings challenge the prevailing belief that severe presentations of babesiosis typically occur in patients over 50, those with severe parasitemia (> 10%), or in immunocompromised individuals, thus adding a unique perspective to the existing literature.

Our patient had no hemoglobinopathies, which could have accounted for his profound hemolysis despite a low parasitemia. He also did not have any history or findings of atrial fibrillation, nor was he taking any prothrombotic medications, which could have otherwise explained his splenic infarctions. A common risk factor in other cases is coinfection with Borrelia burgdorferi. Coinfection with Borrelia burgdorferi or Anaplasma phagocytophilum can increase disease severity and duration [1–4]. Table 3 shows that 5 of 20 cases were associated with other tick‐borne illnesses: 3 with Lyme disease, 1 with Anaplasmosis, and 1 with Ehrlichiosis. Although coinfection may lead to increased severity, many reported cases without coinfection have low parasitemia in immunocompetent patients, suggesting that babesiosis alone can cause spontaneous splenic injury. Thus, babesiosis should not be ruled out as a potential cause of splenic injury, even in the absence of other tick‐borne illnesses.

### 3.3. Pathophysiology of Babesia‐Associated Splenic Injury

The exact mechanism of splenic infarction remains an intriguing enigma. However, a study by Akel et al. [20] demonstrated that inoculating hamsters with Babesia led to microthrombi formation in the spleen’s small vessels, resulting in coagulative necrosis. Infected erythrocytes triggered an acute proinflammatory cytokine release, activating the coagulation system and increasing erythrocyte adhesion. This sequence of events obstructed flow and caused congestion in the splenic sinusoids [20]. As seen in malaria, infected RBCs cannot deform and thus further obstruct splenic sinusoids [2, 20]. Obstruction can lead to infarction in the affected area. Splenic macrophages also attempt to phagocytose infected RBCs trapped in splenic venules, leading to additional congestion and platelet sequestration, which can result in splenomegaly and potentially, splenic infarction or rupture [2, 21]. Dumic et al. [2] support this theory by describing findings of red pulp hyperplasia, diffuse hypercellularity, massive proliferation of plasma cells, and remarkable intrasinusoidal histiocytes in the ruptured spleens of patients infected by Plasmodium vivax.

Table 3 indicates that babesia‐associated splenic injury can occur independent of age, sex, immune status, comorbidities, or degree of parasitemia. However, splenic injuries are more likely in cases where there was a delay in presentation/treatment after the onset of symptoms. The finding that 53% of those cases (see Table 3 and Dumic et al. [2]) where babesiosis was initially missed resulted in worse outcomes further supports this theory. This likely stems from prolonged exposure to a heightened proinflammatory state as described by Akel et al. [20].

In the case of our patient, another factor that may have contributed to his condition was the fall he experienced before presentation. He found the tick before the fall, but it is unclear how long it had been attached before it was first noticed. One may argue that, in the same way that infectious mononucleosis makes the spleen more susceptible to damage upon impact, the same may be true in babesiosis. Hence, the fall and impact on an already compromised spleen from subclinical babesiosis at that point could have been another factor that contributed to the development of his splenic infarcts.

### 3.4. Importance of Early Diagnosis and How Delays can Affect Clinical Outcomes

Timely diagnosis is pivotal in decreasing disease‐related morbidity and mortality. Of the 38 known cases (including our case) of babesia‐associated splenic injury, 19 (50%) were initially missed. 10 out of the 19 cases (53%) had poor outcomes (death or required splenectomy/splenic artery embolization). 2 out of those 10 cases resulted in death, while 8 required splenectomy or splenic artery embolization. The remaining 9 cases were successfully treated with medical management, like our patient. Of those 9 cases, the patients either had no comorbidities or only hypertension. Dumic et al. [6] and Mateja et al. [13] described patients with splenic rupture who required emergency surgery and were discharged, only to return later with postoperative fever, eventually diagnosed with babesiosis as the cause of their splenic rupture.

Our patient initially presented with abdominal pain and splenic infarcts at an outside hospital, where he was discharged afebrile and stable. He later represented with fever, anemia, and more extensive splenic infarcts. This case underscores the necessity of maintaining a high suspicion for babesiosis in patients with atraumatic splenic injury or splenic injury out of proportion to the level of trauma, living in or who have recently traveled to endemic regions. Taking a detailed history of tick exposure and insect bites in endemic areas is crucial to prevent delays in diagnosis and treatment, which can lead to worse outcomes.

### 3.5. Treatment Pearls

Atovaquone and azithromycin treatment is as effective as clindamycin and quinine, with fewer adverse reactions [23]. In a randomized trial by Krause et al. [23], adverse reactions occurred in 15% of patients treated with atovaquone and azithromycin, compared to 72% for those given quinine and clindamycin.

Resistance to atovaquone–azithromycin has been observed in severely immunocompromised patients, limiting its use [24]. Additionally, due to insufficient data on atovaquone–azithromycin efficacy in immunocompromised hosts with severe babesiosis, quinine, and clindamycin are recommended in such cases [23]. For patients with high‐grade parasitemia > 10% (regardless of the clinical presentation), severe anemia (< 10 g/dL), or significant complications (with any level of parasitemia), RBC exchange is used alongside drug therapy [1, 2, 23].

## Consent

No written consent has been obtained from the patient, as there are no patient‐identifiable data included in this case report.

## Conflicts of Interest

The authors declare no conflicts of interest.

## Funding

No funding was received for this manuscript.

## Data Availability

The data that support the findings of this study are available from the corresponding author upon reasonable request.
